# Thermal Degradation Kinetics of Sugarcane Bagasse and Soft Wood Cellulose

**DOI:** 10.3390/ma10111246

**Published:** 2017-10-28

**Authors:** Samson M. Mohomane, Tshwafo E. Motaung, Neerish Revaprasadu

**Affiliations:** Department of Chemistry, University of Zululand (KwaDlangezwa Campus), Private Bag X1001, KwaDlangezwa 3886, Kwazulu Natal, South Africa; samsonmohomane@gmail.com (S.M.M.); RevaprasaduN@unizulu.ac.za (N.R.)

**Keywords:** lignocellulosic waste, kinetics modelling, apparent activation energy, thermogravimetric analysis

## Abstract

The properties of untreated sugar cane bagasse (SCB) and soft wood (SW) and their respective celluloses were investigated. The celluloses indicated improved crystallinity index values and decreased concentration of lignin and hemicellulose compared to their untreated counterparts. Three degradation models, Kissinger-Akahira-Sunose (KAS), Flynn-Wall-Ozawa (OFW), and Kissinger (KGR) methods were employed to determine apparent activation energy values. Generally, the thermal degradation processes of both sugarcane bagasse and soft wood included dehydration, degradation of hemicellulose and cellulose, whereas the lignin degraded from the degradation temperature of hemicellulose to the end of the cellulose. The apparent activation energy values obtained from the OFW and KAS models vary with the degree of conversion, and showed similar trends. The activation energies obtained by KGR were relatively lower than those obtained from the KAS and OFW methods.

## 1. Introduction

Recently a considerable interest has been shown in the utilization of natural fibers as reinforcing fillers in thermoplastic composite materials. The unique properties of natural fibers include low density, good specific modulus values, considerable toughness, flexibility, easy processing, non-toxicity, non-abrasion during processing, recyclability, and resistance to corrosion. Lignocellulosic fibers from agricultural residues/biomass mainly consist of natural composites of polymers (cellulose, hemicellulose, and lignin) [1,2]. Sugarcane is one of the most abundant biomasses in tropical countries such as Brazil and India, creating about 54 million dry tons of residue bagasse (SCB) per year. SCB typically contains approximately 26.6%–54.3% cellulose, 22%–32% hemicelluloses, and about 14%–25% lignin, as well as small proportions of ash (2%–4%) and waxes [3,4,5]. A handful of farmers around “uMhlathuze” municipality in South Africa also produce sugar cane, which is mainly used as an energy source, though the surplus of SCB is much higher than demands. The case of wood chips surplus around the municipality is worse. Industries around, which process wood, face a surplus of chips that end up abandoned in the environment. Wood chips are complex biomass material composed of polysaccharide cellulose, hemicellulose, lignin, and small proportions of extractives. Soft wood (SW) is widely used in buildings, furniture, and paper pulp production [6,7,8]. Since the common practice is to burn the biomasses, it would be worthwhile to obtain more insight into the thermal degradation kinetics of the biomass. Knowledge of kinetic parameters, such as the reaction rate and activation energy, is one of the keys to determine reaction mechanisms in solid phases. Solid state kinetic data are of major and growing interest in many technological processes. These processes include, for instance, thermal decomposition of crystalline solids and energetic materials, thermal oxidation and decomposition of polymers and coal, crystallization of glasses and polymers, and pyrolysis and combustion of biomass resources [9,10].

The thermal degradation kinetics of SCB and SW fibers have been investigated using thermogravimetric analysis (TGA) at various heating rates. Detailed kinetic analysis of the degradation process were performed using model based isoconversional methods to predict the kinetic parameters, i.e. pre-exponential factor, A, and activation energy, E_a_. Motaung et al. [11] and Edries et al. [12] investigated the thermal degradation kinetics of alkali treated SCB and SCB chars, respectively. The alkali treated sample showed the highest values of activation energy in all the investigated degree of conversions, as compared to sulfuric acid treated SCB. The authors observed that the peak of weight loss rate in the differential thermogravimetry (DTG) curves shifted to a higher temperature with increasing heating rate. Ramajo-Escalera et al. [10] applied Vyazovkin’s model-free kinetics to determine conversion, isoconversion, and apparent activation energy of SCB and detected three steps. The apparent activation energy values were 76.1 ± 1.7 kJ·mol^−1^, 333.3 ± 15.0 kJ·mol^−1^ and 220.1 ± 4.0 kJ·mol^−1^ in the conversion range of 2%–5%, 15%–60% and 70%–90%, respectively.

Thermal degradation kinetics of wood has also been given attention by many researches using the Kissinger-Akahira-Sunose, Flynn-Wall-Ozawa, and Coats-Redfern methods [13,14,15,16,17,18]. The results showed that the whole course of pyrolysis of wood can be divided into three phases and the activation energy results of the Kissinger method are higher than Kissinger-Akahira-Sunose. The objective of this paper is to systematically investigate and compare the thermal degradation kinetics of SCB and SW celluloses under similar preparation conditions. Various degradation models including the Kissinger-Akahira-Sunose, Flynn-Wall-Ozawa, and Kissinger methods were used to determine the apparent activation energy values.

## 2. Material and methods

### 2.1. Materials

Sugar cane bagasse and soft wood were collected from industries respectively located near Empangeni, South Africa. Sodium hydroxide pellets (99.9%) were supplied by Merck, Mumbai, India, Sodium perchlorite by Capital lab Suppliers CC, New Germany, Durban, South Africa. Glacial acetic acid was supplied by Minema Chemicals, Roodepoort, South Africa. All chemicals were used as received without further purification.

### 2.2. Chemical Composition

The untreated SCB and SW samples were used to determine their respective chemical composition. The lignin content was analyzed according to a standard method recommended in TAPPI-T222 om-88 [19] and the cellulose content obtained as described in TAPPI T19m-54 standards [19].

### 2.3. Extraction of SCB and SW Cellulose

The SCB and SW samples were washed and immersed in water bath for 24 h at room temperature before drying at 60 °C overnight. Then SCB and SW were treated with an alkali solution (2 wt % NaOH) at 100 °C for 4 h. The solid precipitates were then filtered and washed several times using distilled water to pH neutral. This treatment was performed four times. The same treatment was performed using 1.7 wt % sodium hypochlorite buffered with acetic acid. Finally the suspension was allowed to cool and rinsed using deionized water to neutral pH.

### 2.4. Characterization Methods

The FTIR spectra were collected by using a Perkin Elmer FTIR spectrometer (Stillwater, GA, USA) in the diffuse reflectance mode. The samples were analyzed in the spectral region between 4000 and 400 cm^−1^ with a 4 cm^−1^ resolution.

Powder X-ray diffraction (XRD), (Bruker AXS D8 Advance X-Ray diffractometer, Karlsruhe, Germany) equipped with Cu Kα generator (λ = 0.154 nm) as X-ray source, operating (40 kV, 40 mA) was used to examine the crystal structure of SCB, SW and extracted celluloses.

Thermogravimetric analyses were performed using a TGA analyzer unit (Perkin Elmer, Foster City, CA, USA), under a flowing nitrogen atmosphere at a flow rate of 20 mL·min^−1^. Approximately 10 mg of sample was heated from 25 to 600 °C, at different heating rates rate of 3 °C·min^−1^, 7 °C·min^−1^, 11 °C·min^−1^, and 15 °C·min^−1^. The sample weight loss and rate of weight loss were recorded continuously as functions of temperature.

### 2.5. Degradation Kinetics

Different kinetic models are proposed to understand degradation mechanisms through prediction of the kinetic parameters based on the data obtained from TGA curves. The kinetic parameters (i.e., A, n, and E) can be calculated from TGA data by using the following rate equation [20,21,22,23]:

dα/dt = k(T)·f(α)
(1)

where α represents the extent of reaction, which is determined from the TGA data (fractional mass loss), t is time, k(T) represents the temperature dependent rate constant expressed by an Arrhenius type expression, and f(α) denotes the particular reaction model, which determines the dependence of the reaction rate on the extent of reaction. In this study, the conversion rate is defined as:

α = (W*_o_* − W*_t_*)/(W*_o_* − W*_f_*) (2)

where W*_t_*, W*_o_*, and W*_f_* are time t, initial and final weights of the sample, respectively. The temperature dependence of the rate constant can be expressed by the Arrhenius equation:

k = A exp ((−E)/RT)
(3)

where A and E are pre-exponential factor and activation energy, respectively. Equation (1) can be written as

dα/dt = A exp ((−E)/RT)f(α)
(4)

In order to determine the kinetic triplet (A, E, and f(α)), various methods have been developed. They are classified as isoconversional and model fitting methods. In this study Kissinger-Akahira-Sunose, Flynn-Wall-Ozawa, and Kissinger models were considered for determining the kinetics of the thermal degradation of SCB and SW celluloses.

#### 2.5.1. Flynn Wall Ozawa (OFW) Model

OFW model is an integral isoconversional method derived by using Doyle’s approximation using multiple heating rate TGA data. The model expression is given by

ln β = ln AE/f(α)R − 2.315 − 0.4567 E/RT
(5)

The plot of ln β vs. 1/T gives a straight line whose slope is equal to –0.4567E/RT from which the activation energy can be calculated. The pre-exponential factor is calculated from the intercept of the resulting straight line by assuming a reaction model.

#### 2.5.2. Kissinger Akahira Sunose (KAS) Model

KAS is an isoconversional model based on the numerical approximations of the Arrhenius integral over a wide range of thermal history. The model expression is written as

ln (β/T^2^) = ln (AR/(Ef(α))) − E/RT (6)

The plot of ln (β/T^2^) vs. 1/T yields a straight line. The values of E and A can be calculated from the slope and intercept for a particular reaction model.

#### 2.5.3. Kissinger (KGR) Model

This is a maximum rate method and applicable only to the multiple heating rate TGA or DTG data. The temperatures (T_m_ or T_p_) at which the rates reach maximum weight loss are used to predict the single values of E_a_ and A. In the Kissinger method, ln (β/T_m_^2^) is plotted against 1/T_m_ for a series of experiments at different heating rates with the maximum peak temperature, T_m_, obtained from the DTG curve.

The expression for this model is

ln (β/T_m_^2^) = −(E/(RT_m_)) + ln (AR/E) (7)

## 3. Results and Discussion

### 3.1. Chemical Compositions of SCB and SW

The chemical composition of sugarcane bagasse and soft wood are shown in Table 1. As expected, the cellulose content in both untreated sugar cane bagasse and the wood was the highest. However, the wood revealed an uncommon behavior in which hemicelluloses was dominated by lignin content. There are lot of factors involved for the results including the method used to measure chemical compositions and the origin of the cell wall structure [3,4,5,10].

### 3.2. Structure Characterization

**Figure 1:**
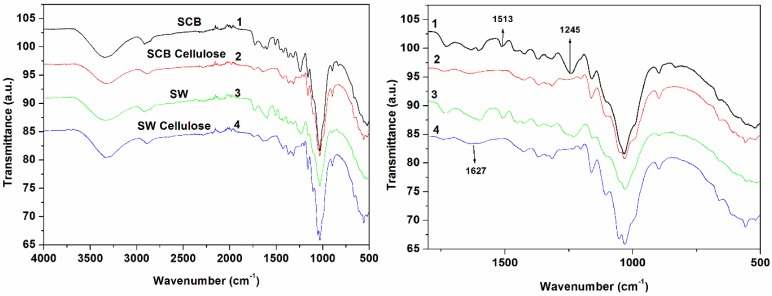
FTIR spectra of untreated SCB, SCB cellulose, untreated SW, and SW cellulose.

FTIR spectra of SCB, SCB cellulose, SW, and SW cellulose are shown in Figure 1. The untreated SCB and SW materials showed similar patterns with the dominant peaks observed at approximately 3340 cm^−1^ (O–H stretch), 2892 cm^−1^ (C–H vibrations), 1730 cm^−1^ (C=O stretching), 1627 cm^−1^ and 1513 cm^−1^ (C=C aromatic), 1245 cm^−1^ (O-H vibration of phenolic group), 1110 cm^−1^ (C–O–C stretching), 1051 cm^−1^ (O–H stretching), and 897 cm^−1^ (β-glycosidic linkage). These peaks are typical for lignocellulosesic materials and are known for sugar cane bagasse and soft wood [6,7,24,25,26,27]. However in this study the wood has blunt peaks between 1000 and 2000 cm^−1^, which may explain the difference in chemical compositions. The peaks at 1241 cm^−1^ and 1722 cm^−1^ in the celluloses, normally linked to aromatic skeletal vibrations of lignin and hemicelluloses, were reduced and others are almost invisible as compared to untreated counterparts [8]. This indicated removal of lignin and hemicelluloses, as well as an exposure of cellulose [28].

### 3.3. Crystallinity Characterization

**Figure 2:**
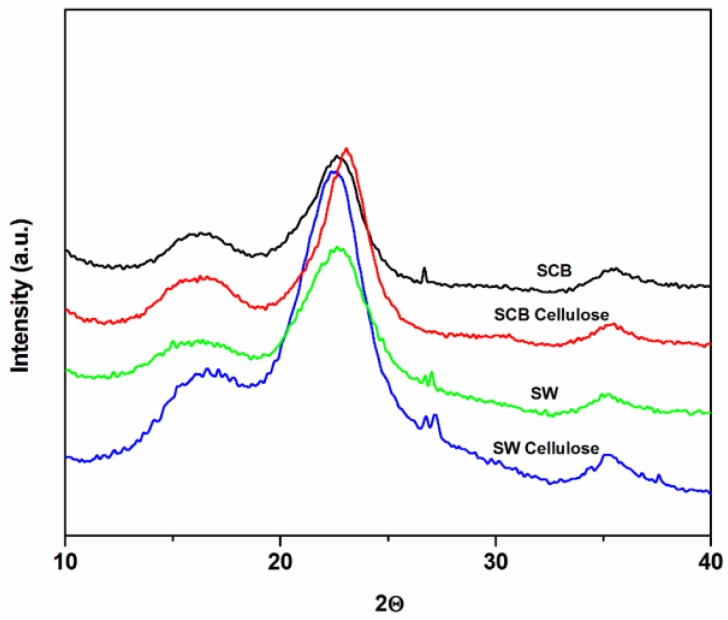
XRD analysis of untreated SCB, untreated SW, SCB cellulose, and SW cellulose.

Figure 2 and Table 2 showed XRD analysis to evaluate the crystallinity of SCB and SW materials. The diffraction pattern of untreated SCB and SW indicated typical behavior of cellulose I type, which showed major intensity peaks related to crystalline structure at 2*θ* values of around 15.5° and 22.5° [4,8,21]. The Segal and deconvolution methods were used to calculate crystallinity index values of the samples respectively. For the first method crystallinity index (CI) was obtained from the ratio of the maximum peak intensity 002 (I_002_, 2*θ* = 22.5) and halo depression (I_am_ 2*θ* = 18.5) between peaks 001 and 002 according to Equation (8).

(8) $$\mathrm{CI}\left(\%\right)=\frac{I_{002}-I_{\mathrm{am}}}{I_{\mathrm{am}}}\times 100$$

where I_002_ is the maximum intensity of the 002 peak and I_am_ the minimal depression of the amorphous structure.

For the second method, individual peaks were fitted by Gaussian functions to predict areas according to the following Equation (9).

(9) $$\mathrm{CI}\left(\%\right)=\frac{\Sigma \;A_{\text{cryst}}}{\Sigma \;A_{\text{cryst}}+\Sigma \;A_{\text{amorp}}}\times 100$$

where A_cryst_ and A_amorp_ are the fitted areas of the crystal and amorphous domains, respectively.

The untreated materials presented the lowest relative crystallinity index, because of higher content of amorphous hemicellulose, and lignin. The treatment of NaOH and bleaching agents removed the lignin and hemicellulose content and increased the degree of crystallinity index by almost 20%. The effect is well known in the literature [29,30]. In this study the effect corresponded to the chemical composition. This is underlined by the fact that the highest containing cellulose of untreated material has the highest crystallinity compared to the untreated counterparts. However after the treatment, SW cellulose surpassed SCB cellulose by 30%. This confirms the removal of lignin and hemicellulose as alluded to by FTIR results. Nonetheless, the difference in crystallinity index values obviously arose from the difference in compactness of the cell walls [3,4,5], which responded differently to cellulose extractions.

### 3.4. Thermal Stability

**Figure 3:**
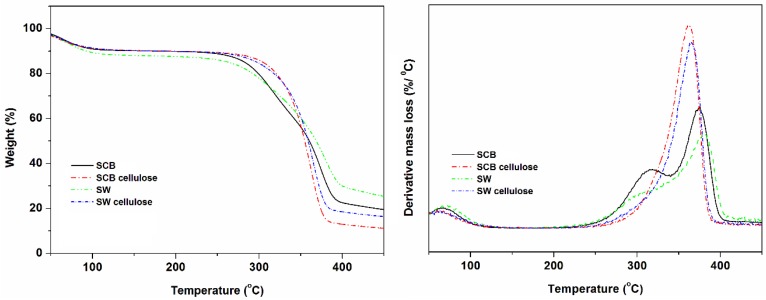
TGA and DTG graphs of SCB, SW, SCB cellulose, and SW cellulose at 11 °C·min^−1^.

Figure 3 represented the TGA and DTG curves of untreated SCB, untreated SW, SCB cellulose, and SW cellulose at 11 °C·min^−1^. The untreated materials display three degradation stages as confirmed by DTG, and SW is thermally more stable than SCB with a higher char content than the rest. On the other hand, both celluloses display two degradation stages with virtually similar higher thermal stability than untreated counterparts. The celluloses generally have lower char contents than untreated materials; however SW cellulose dominates SCB cellulose content. The first degradation stage is normally attributed to a release of moisture, whilst the second and the third stage, in the case of untreated materials, are attributed to degradation of lignin, hemicelluloses, and cellulose [31,32,33,34,35]. The removal of lignin and hemicelluloses as confirmed in Fourier transform infrared spectroscopy (FTIR) and XRD results showed both celluloses with two stages for water release and cellulose scission. As for the difference in char content, Moniruzzaman et al. [8] and Shen et al. [14] related a similar observation to the removal of cellulose, the origin of the lignin, and the cell wall. In this study, the reactive hypochlorite treatment, which seemed to have altered the chemistry of both celluloses to form different derivatives could also be responsible for the difference in char content.

**Figure 4:**
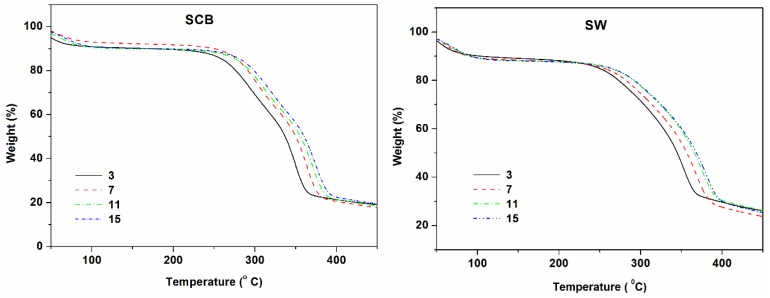
TGA graphs of SCB and SW at different rates.

To obtain more information on thermal degradations, kinetics studies were undertaken using KAS, OFW, and KGR models. Because KGR depends on T_m_, KAS and OFW were compared first, followed by comparison of all models. The models required different heating rates which are known to display increased thermal stability due to the time temperature disposition principle and the particle thermal gradient theory of lignocellulosic materials [14,36]. All the current results obeyed the principle within experimental uncertainty as shown by exemplary graphs of untreated SCB and SW (See Figure 4).

### 3.5. Kinetics of Degradation

**Figure 5:**
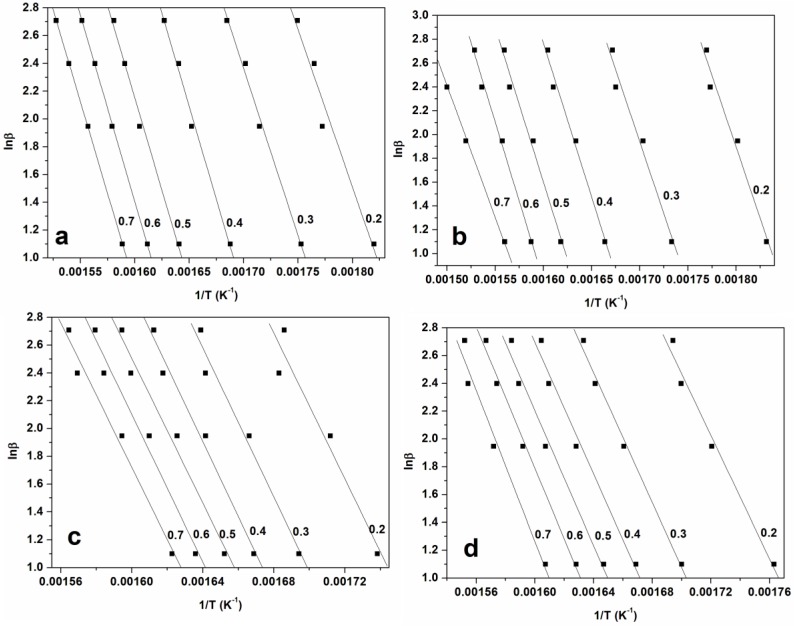
(**a**) FWO of untreated SCB; (**b**) FWO of untreated SW; (**c**) FWO of SBC cellulose; (**d**) FWO of SW cellulose.

**Figure 6:**
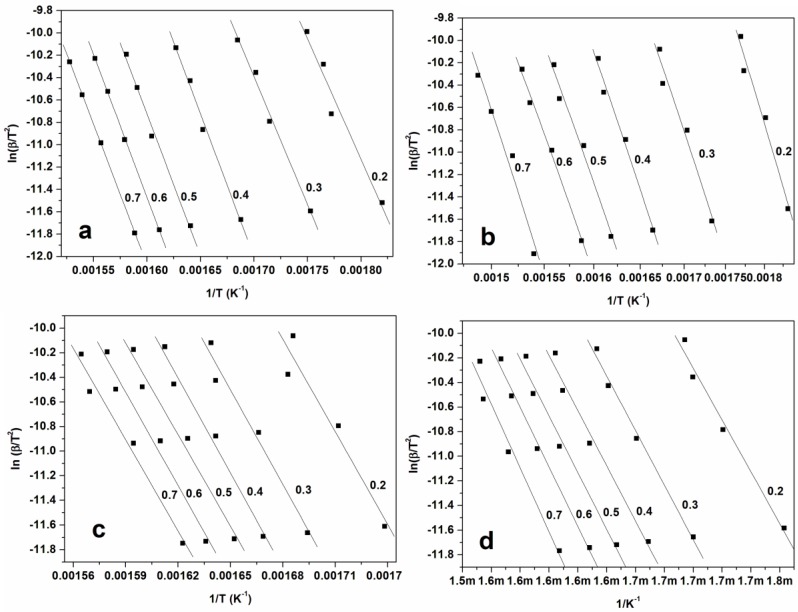
(**a**) KAS of Untreated SCB; (**b**) KAS of untreated SW; (**c**) KAS of SBC cellulose; (**d**) KAS of SW cellulose.

**Figure 7:**
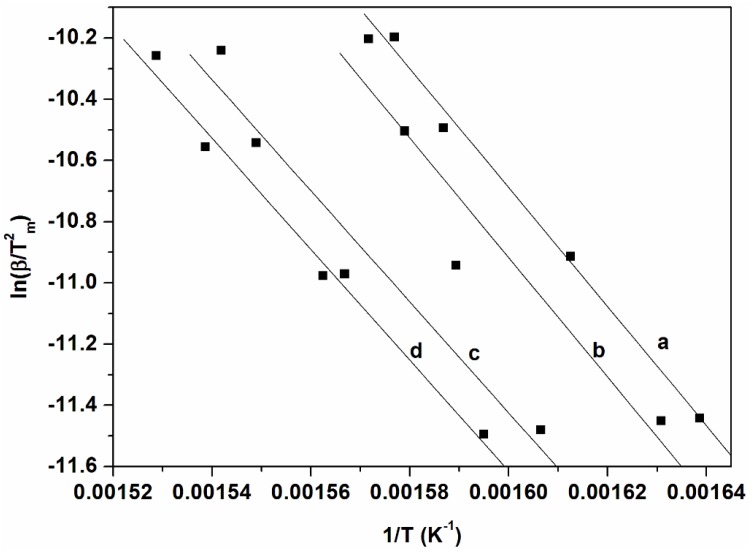
(**a**) Kissinger of untreated SCB; (**b**) Kissinger of SBC cellulose; (**c**) Kissinger of untreated SW; and (**d**) Kissinger of SW cellulose.

**Figure 8:**
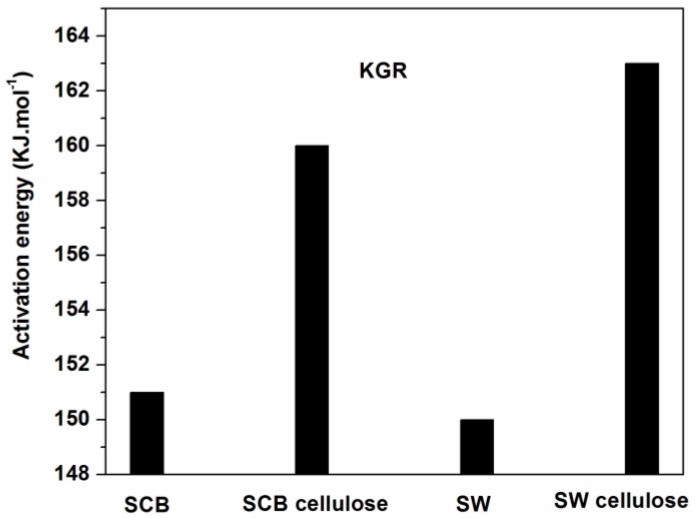
Activation energy values as a function of degrees of conversion obtained by KGR.

**Figure 9:**
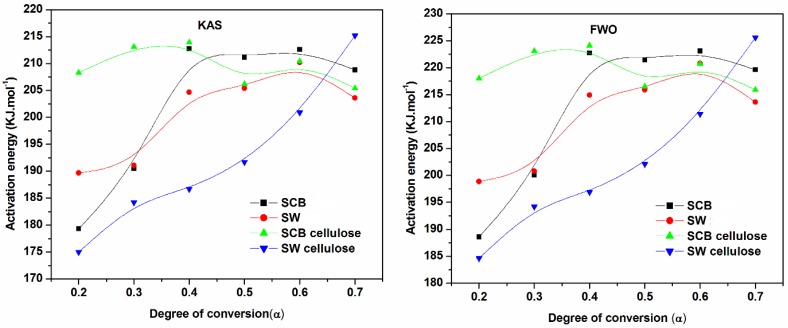
Activation energies (kJ mol^−1^) obtained by KAS and FWO methods. LG: levoglucosan, HAA: hydroxyacetaldehyde, HA: hydroxyactone, PA: pyruvic aldehyde, GA: glyceraldehyde, 5-HMF: 5-hydroxymethyl-furfural, FF: furfural, and AGF: 1,6-anhydro-β-glucofuranose.

The plots of ln β and ln (β/T^2^) against 1/T corresponding to the several conversion degrees are shown in Figure 5, Figure 6 and Figure 7 for FWO, KAS, and KGR respectively. The Kissinger model unaided was also employed as presented in Figure 8, which showed linear plots of ln (β/T_m_^2^) versus 1/T_m_. The activation energy values were calculated from the slopes of the isoconversional plots. It can be observed in all cases that the lines becomes nearly parallel indicating the possibility of a simple reaction mechanism (Figure 6 and Figure 7 for all a,b,c, and d). Similar observations were also reported in the literature and our activation energy values were within the range of reported values (110–300 kJ·mol^−1^) [1,10,21]. However in this study it is worth noting that the gap between the lines is generally wider for untreated materials compared to cellulose (Figure 6a,b and Figure 7a,b). Furthermore, the slopes are generally steeper for all untreated materials compared to celluloses. These might probably relate to the accelerated decomposition process of the main degradation stage in the untreated materials as compared to the delayed process in celluloses. Figure 9 showed the relationship between activation energy (E_a_) values calculated from the corresponding slopes of the degree of conversions. The overall trend of E_a_ was relatively similar for both SCB and SW materials, from which there was an increase in the degree of conversion from 20%–50% followed by a gentle reduction up to 70% mass loss. The SCB cellulose revealed a slight decrease in activation energy while SW showed a linear proportional relationship with the increase in the degree of conversion.

**Figure 10:**
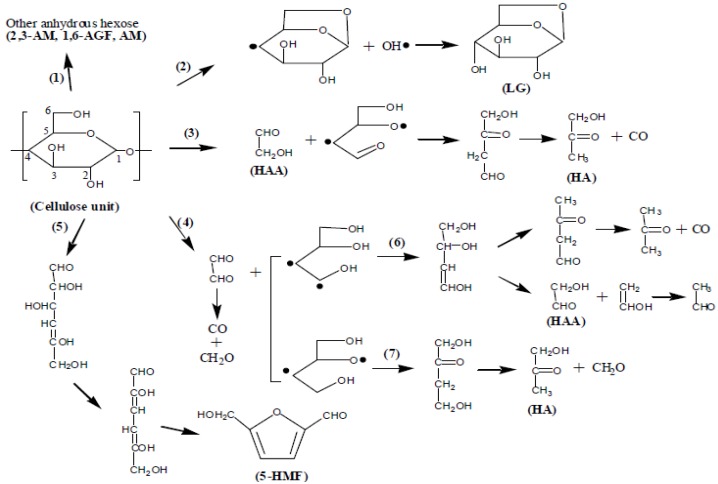
The speculative chemical pathways for the direct conversion of the cellulose molecules.

In the reported trends in previous research, hemicellulose and cellulose of lignocellulosic materials degrade at lower and higher temperatures respectively, while lignin degrades across the temperature ranges [37,38]. This suggests in our case that the most char for untreated materials formed at 70%. The cellulose apparently decomposes into volatile levoglucosan and glycolaldehyde which then interact exothermically to form a char. Shen et al. [14] proposed the pathways as indicated in Figure 10.

The initial thermal degradation of cellulose is the depolymerization of the cellulose polymer to form various anhydrosugar derivatives including levoglucosan as the most prevalent. It can be seen from the mechanism that levoglucosan (LG) is reduced at higher temperature, in contrast to other products which implies a complex and competitive mechanism between the LG and other products (such as HAA, HA, and 5-HMF). In our case it is clear that initially treatments led to completely different cellulose derivatives which rendered a higher activation value for SCB cellulose than SW cellulose at 20% mass loss. However at 70% mass loss SW cellulose has the highest activation value that corresponds to its char content which is twice that of SCB cellulose. This seems to agree with Pathway 5 which indicated formation of 5-hydroxymethyl-furfural which can form a crosslinked structure to increase char content once polymerized. This in fact could explain the different behaviors of activation energy values for the celluloses. The Kissinger model indicated that both celluloses displayed higher activation energy values than their corresponding untreated counterparts, even though the SW cellulose slightly surpassed that of SCB cellulose (Figure 8). This clarifies the observed increased in thermal stability of celluloses and is in line with the increased crystallinity index values of celluloses.

## 4. Conclusions

Thermal degradation of SCB and SW was investigated using TGA at different heating rates of 3 °C·min^−1^, 7 °C·min^−1^, 11 °C·min^−1^, and 15 °C·min^−1^. The thermal degradation of SCB and untreated SW materials displays a three stage weight loss, which corresponds to the three peaks on the DTG curves: moisture evaporation, decomposition of hemicellulose, and cellulose, and decomposition of lignin content. Increasing the heating rate resulted in increasing mass loss rates, but the start of thermal decomposition was delayed to higher temperatures. The thermal stability of extracted cellulose increased and the high content of lignin produces more char residue for the untreated counterparts. Kinetic parameter in terms of the apparent activation energy were determined and compared using the FWO, KAS, and KGR methods. The apparent activation energy of both FWO and KAS methods varied with conversion fractions, and showed similar trends. The FWO method provided higher activation energy values than those from the KAS method. The Kissinger model resulted in lower apparent activation energy than was obtained using the other kinetic models.

## Figures and Tables

**Table 1:** Fractions of principal constituents of SCB and SW.

Sample	Lignin (%)	Cellulose (%)	Hemicellulose (%)
SCB	18.8 ± 0.1	42.9 ± 0.99	38.2 ± 1.08
SW	37.2 ± 0.01	39.2 ± 0.01	23.6 ± 0.01

**Table 2:** Crystallinity index of u SCB, SW, SCB cellulose and SW cellulose.

Samples	Segal Method	Deconvolution Method
SCB	53.9	33.5
SW	52.8	32.3
SCB cellulose	61.3	43.9
SW cellulose	88.7	57.3
